# Ultrasonography of the Tympanic Bulla in Llamas and Alpacas: Techniques and Physiological Findings

**DOI:** 10.3390/ani15121762

**Published:** 2025-06-14

**Authors:** Rainer Giebl, Johann Maierl, Alexander Tichy, Cassandra Eibl, Agnes Dadak, Sonja Franz

**Affiliations:** 1 Clinical Department for Farm Animals and Food System Science, Centre for Ruminant and Camelid Medicine, University of Veterinary Medicine Vienna, Veterinärplatz 1, 1210 Vienna, Austria; giebl@aon.at (R.G.); cassandra.eibl@vetmeduni.ac.at (C.E.); 2 Veterinary Faculty, Department of Veterinary Sciences, Chair of Anatomy, Histology and Embryology, Ludwig-Maximilians-University Munich, Veterinärstrasse 13, 80539 Munich, Germany; j.maierl@anat.vetmed.uni-muenchen.de; 3 Department of Biological Sciences and Pathobiology, Centre of Biological Sciences, University of Veterinary Medicine Vienna, Veterinärplatz 1, 1210 Vienna, Austria; alexander.tichy@vetmeduni.ac.at; 4 Department of Biological Sciences and Pathobiology, Centre of Biological Sciences, Pharmacology and Toxicology, University of Veterinary Medicine Vienna, Veterinärplatz 1, 1210 Vienna, Austria; agnes.dadak@vetmeduni.ac.at

**Keywords:** otitis media, ear, examination, diagnosis, South American camelids

## Abstract

This study aimed to perform ultrasonography of the tympanic bulla in healthy, non-sedated camelids for the first time. In the first step, dissection and sonography in cadaver specimens allowed the essential identification of the anatomical structures of the region of interest. Ultrasonography of the tympanic bulla was then performed on 71 healthy llamas and alpacas, and the length of the visible lateral bulla wall was measured sonographically. The reproducibility of measuring was determined between two examiners. Differences in length and the influences of gender, age, and body condition scores were monitored. A 10 MHz linear probe was positioned in the rostrocaudal and dorsoventral directions caudal to the mandibular ramus and ventral to the base of the ear using a lateral approach. The equipment used proved to be appropriate to visualize the tympanic bulla, the zygomatic bone, the paracondylar process, the external ear canal, and the parotid gland in the cadaver specimens and all live animals. Llamas revealed a significantly longer visible lateral bulla wall than alpacas, but its length was not influenced by gender, body condition score, or age. In alpacas, older females showed a significantly shorter tympanic bulla wall. The intra- and inter-observer reliability yielded a significant agreement. Based on this study’s results, ultrasonography may serve as a non-invasive diagnostic tool for detecting osseous and soft-tissue changes in camelids with otitis media.

## Simple Summary

Computed tomography and radiography are considered the “gold standard” for visualizing the tympanic bulla and the diagnosis of otitis media in camelids. Both methods must be performed in a sedated or anesthetized animal. Ultrasonography, a non-invasive technique, requires no sedation or anesthesia of the animal. This study aimed to evaluate the ultrasonographic examination of the tympanic bulla and describe the sonographic approach and appearance of the visualized structures in healthy camelids for the first time. A 10 MHz linear probe was positioned caudal to the mandibular ramus and ventrally to the base of the ear using a lateral approach, both in the rostrocaudal and dorsoventral directions. The equipment used proved to be appropriate to visualize the tympanic bulla and surrounding structures in cadaver specimens and healthy live camelids. Measuring the visible lateral part of the bulla wall revealed that llamas have a significantly longer bulla wall than alpacas. The intra- and inter-observer reliability between two ultrasonographers measuring the bulla length yielded a significant consistency. The results of this study demonstrate the feasibility of ultrasonographic imaging to visualize the tympanic bulla in healthy camelids. Further studies evaluating the diagnostic potential of ultrasonography in llamas and alpacas suffering from otitis media are required.

## 1. Introduction

Otitis media in South American camelids (SACs) develops either from the extension of the otitis externa or, more often, following ascending bacterial infection via the eustachian tube [1]. In llamas and alpacas, otitis media can be subclinical or clinically apparent by developing a head tilt, with or without trigeminal and facial nerve deficits, such as a dropped ear, ipsilateral flaccid facial muscles, and saliva, as well as food dripping out from the mouth. Otic discharge is observable in only a few cases. Vestibular signs, such as circling or nystagmus, can develop in severe cases [1,2]. Treatment at an early stage is greatly important for clinical outcomes; otherwise, prognosis is very poor [1]. Early diagnosis, therefore, can prevent the development of chronic pathological changes, such as abscessation and bone sequestration within the tympanic bulla or even death of the animal [1,2,3,4,5,6,7,8]. Diagnostic imaging techniques, such as radiography or computed tomography (CT), are currently considered the “gold standard” for the visualization of the anatomic structures of the middle and inner ear and, therefore, for the diagnosis of otitis media. Both methods must be performed in a sedated or anesthetized animal [1,2,6,9,10].

Ultrasonography is frequently used in veterinary medicine for various indications and usually requires no sedation or general anesthesia of the patient. Pathological changes in soft tissues (e.g., inflammation, fibrosis, steatosis, abscessation, and calcification) and bone surfaces (e.g., fractures, osteolytic processes, and sequestration) can be detected. Its great advantage is its non-invasive character, which makes ultrasound applicable in the field as well [11,12].

Ultrasonography has already been performed for the visualization of the tympanic bulla in various animal species [13,14,15,16,17,18,19,20]. Dickie et al. demonstrated the feasibility of ultrasonography of the canine tympanic bulla in cadaver specimens and non-sedated healthy live dogs [13]. The sonographic imaging and description of the tympanic bulla’s sonographic appearance was also successfully performed in feline, calf, and rabbit cadaver specimens [16,17,21], as well as in live calves and rabbits [16,20]. Sharsher et al. investigated this technique in healthy donkeys, emphasizing its applicability to this animal species [19]. The diagnostic potential of ultrasonography was evaluated in live calves suffering from otitis media. As a confirmatory examination technique, a histological examination was used, representing a low sensitivity, ranging from 32 to 63%, and a high specificity, ranging from 84 to 100% [22]. In diseased dogs, ultrasonography showed a comparable sensitivity of 37–74% and a lower specificity of 55–74% compared with CT [23,24].

There are no descriptions of the ultrasonography of the tympanic bulla in llamas and alpacas. This study hypothesized that ultrasonography of the tympanic bulla can also be successfully performed in SACs, but the technique and sonographic physiological findings will differ due to specific anatomical differences. The external ear canal is divided into a lateral–vertical cartilaginous canal and a short and narrow bony horizontal canal that, in turn, is separated from the bony honeycomb-structured tympanic bulla by the tympanic membrane [6,9,10,25]. The tympanic bulla extends ventrally of the external ear canal in the transverse plane as a narrow, almost rectangular structure from just under the skin to the base of the skull [25]. Hence, this study aimed to describe the method and normal sonographic appearance of the tympanic bulla and its surrounding structures in cadavers and healthy, non-sedated alpacas and llamas for the first time.

## 2. Materials and Methods

### 2.1. Animals

In the first part of this pilot study, 2 cadaver heads (adult alpacas: *n* = 1; adult llamas: *n* = 1; no evidence of ear disease) were dissected ventral to the base of the left ear to identify the anatomical structures of the region of interest and determine anatomical landmarks for ultrasonographic examination. Furthermore, the normal sonographic anatomy and appearance of the tympanic bulla and surrounding structures were described in 4 additional cadaver head specimens (left side of the head) (adult alpacas: *n* = 2; adult llamas: *n* = 2; no evidence of ear disease), establishing a scanning technique and comparing the sonograms between animal species.

In the second part of this study, ultrasonography was carried out on live, non-sedated llamas and alpacas of various ages in the field to visualize the tympanic bulla and surrounding structures of the region of interest and measure the sonographically visible length of the lateral edge of the tympanic bulla in the vertical probe position. The animals were selected according to specific inclusion/exclusion criteria as follows: Older than 1 year and used to handling [26]; no history or clinical evidence of an ear disease (otic discharge, head shaking, abnormal position of the pinna, absent ear reflex, flaccid facial muscles, nystagmus, ptosis, atactic, and circling). If female llamas or alpacas were in their late stage of gestation (the last third of gestation), they were excluded from this study. Body condition scores (BCSs) were determined using a scoring system from 1 to 5, where a score of 1 indicated emaciated animals (no fat and a minimal amount of muscle), 3 was considered physiological, and 5 indicated over-conditioned animals with substantial subcutaneous fat depots [27].

### 2.2. Ultrasonographic Examination

Ultrasound examinations and measurements were performed using a portable device (DP-50 Expert^®^, Mindray Medical Germany GmbH, Darmstadt, Germany) with a 10 MHz linear probe (59 mm × 10 mm). In the cadaver specimen, the fleece in the region of interest was shorn, and alcohol (70% ethanol) was used as a contact medium between the skin and the probe. The linear probe was placed at different positions (in the rostrocaudal and dorsoventral directions) caudal to the mandibular ramus and ventral to the base of the ear to visualize the tympanic bulla and surrounding structures using a lateral approach. A needle attached to a syringe filled with methylene blue helped with identification. The needle was inserted under sonographic control in the same direction as the ultrasound waves to the tympanic bulla, the parotid gland, the zygomatic bone, and the paracondylar process. The tip of the needle could be identified sonographically as a hyperechoic echo producing reverberation artifacts. Methylene blue was injected at the topographic position of the structure to be identified. Correct needle placement was confirmed by dissecting the cadaver head afterward.

Ultrasonography on live animals was performed in non-sedated standing camelids; no restraint chute was used. The head of the llama or alpaca was held by the owner. The same ultrasound machine, probe, and probe positions as those used for the cadaver specimens were used for the live animals. The fleece in the area of interest (the ventral of the base of the ear) was not shorn but was parted with the fingers, and alcohol (70% ethanol) was used as a contact medium between the probe and the skin. Ultrasonography of the left ear was performed from the left body side in all animals. The ability to identify the structures in the rostrocaudal and dorsoventral probe positions in the region of interest was noted, and the length of the lateral edge of the tympanic bulla in the dorsoventral direction was measured using calipers.

### 2.3. Statistical Analysis

All statistical analyses were performed using IBM SPSS v29. Animal species, age, gender, body condition score (BCS), the ability to identify the tympanic bulla and surrounding structures in the live animals, and the length (cm) of the sonographically visible part of the tympanic bulla wall were documented. The data were expressed as means, standard deviations, and ranges. The results of the measured length of the tympanic bulla were compared between the two animal species (llama and alpaca), and the measurements were analyzed regarding the significant differences influenced by the animal species (llama and alpaca), gender (male and female), age, and BCS using a linear model controlling for the covariates of age and BCS.

To evaluate the reproducibility of the ultrasonographic measurement of the length of the tympanic bulla wall, the intra-observer reliability was determined between two examiners who were experienced in performing ultrasonographic examinations using the intraclass correlation coefficient (ICC). Both examiners performed the ultrasonographic measurement independently from each other 3 times in the same animal within 2 h. A total of 16 animals (llamas: *n* = 4; alpacas: *n* = 12), part of the total of 71, were examined. The correlation between the two sets of data (two examiners: A and B) was determined using the Pearson correlation coefficient (r). A *p*-value below 5% (*p* < 0.05) was considered significant for all statistical analyses.

This study was discussed and approved by the Institutional Ethics and Animal Welfare Committee per the GSP guidelines and national legislation. Ethical approval was granted by the Vetmeduni Vienna Ethics Committee (ETK-018/01/2022).

## 3. Results

### 3.1. Identification of Anatomical Structures During Dissection and Ultrasonographic Appearance of Tympanic Bulla and Surrounding Structures in Cadaver Specimens

**Figure 1:**
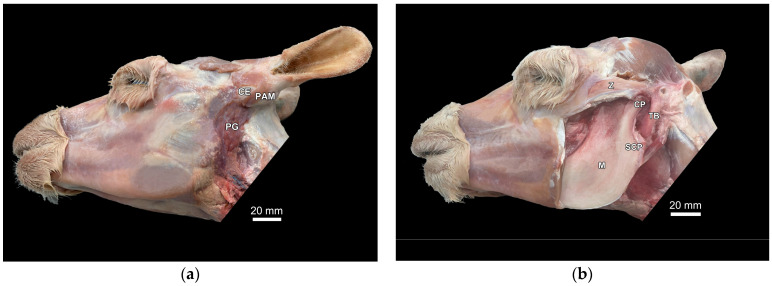
Cadaver dissection in region of interest (area ventral to base of ear) to identify anatomical structures for ultrasonographic examination: Lateral view of a skull of an adult female alpaca (**a**), with skin and muscle layer (cutaneous muscle of face and ventral part of parotidoauricular muscle) removed: parotid gland (PG), dorsal part of parotidoauricular muscle (PAM), and conchal eminence (CE); (**b**) view of bone structures (masseter muscle removed): zygomatic bone (Z), mandibular ramus (M) with condylar (CP) and subcondylar process (SCP), and tympanic bulla (TB).

The area around the tympanic bulla was dissected, both in one llama and one alpaca cadaver head specimen. Figure 1a,b shows an alpaca skull in a lateral view with the relevant anatomical structures of the region of interest during dissection. No differences between the alpaca and llama head cadaver specimens were observed.

In additional cadaver specimens (alpacas: *n* = 2; llamas: *n* = 2), the probe was placed with a lateral approach, ventral to the base of the ear in two positions (the rostrocaudal and dorsoventral positions) to visualize the anatomical structures identified in the dissected cadaver. The detection of ultrasonographic-visible structures was confirmed via the injection of methylene blue, as described in Section 2.

**Figure 2:**
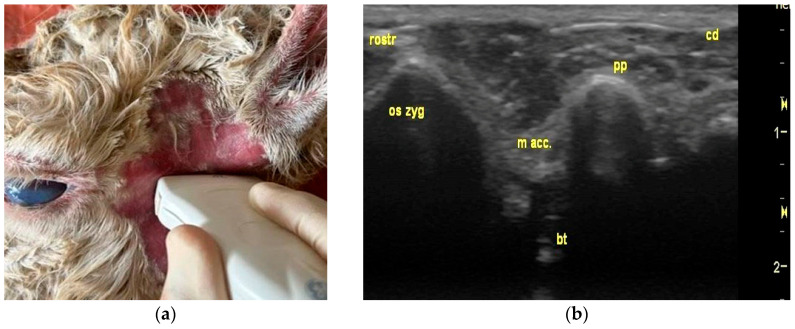
Probe position and ultrasonographic findings in region of interest (left lateral view of a skull of an adult female alpaca). (**a**) rostrocaudal probe position using a lateral approach ventral of base of ear; (**b**) sonogram (rostrocaudal probe position): zygomatic bone (os zyg), external ear canal (m acc.), paracondylar process (pp), tympanic bulla (bt), caudal (cd), and rostral (rostr).

At scan position 1, the scanner was placed in the rostrocaudal position in line with the lateral canthus of the eye, caudal to the vertical mandibular ramus, and ventral to the base of the ear (Figure 2a). In this position, it was possible to sonographically visualize the bony structures, such as the zygomatic bone, the paracondylar process, and the tympanic bulla (Figure 2b). These structures all appeared as a smooth, echoic line with distal acoustic shadowing. In between the zygomatic bone (rostral) and the paracondylar process (caudal), the cartilaginous conchal eminence and a part of the external ear canal were displayed as a hypoechoic structure (Figure 2b).

**Figure 3:**
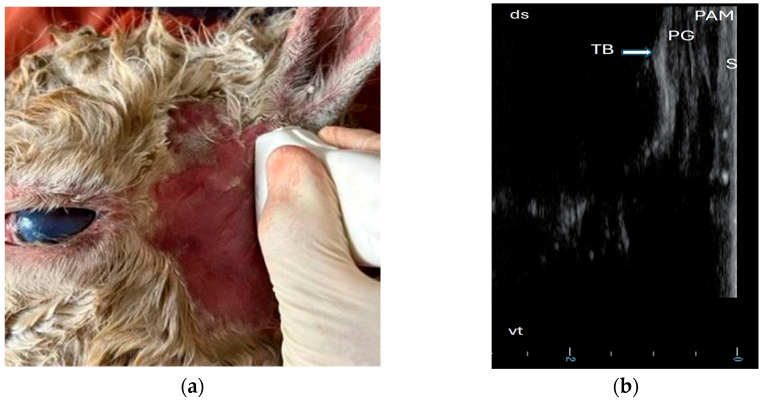
Probe position and ultrasonographic findings in region of interest (left lateral view of a skull of an adult female alpaca). (**a**) dorsoventral probe position using lateral approach ventral of base of ear; (**b**) sonogram (dorsoventral probe position): tympanic bulla (TB), parotid gland (PG), parotidoauricular muscle (PAM), skin (S), dorsal (ds), and ventral (vt).

In scan position 2, the scanner was placed directly ventral to the base of the ear in the dorsoventral direction (Figure 3a). It was possible to visualize the osseous tympanic bulla wall as a smooth, echoic line with acoustic shadowing and reverberation artifacts, representing the lateral edge of the tympanic bulla. The ventral end exhibited a convex shape, corresponding to the ventral medial part of the tympanic bulla. The lateral tympanic bulla wall was covered by a homogenous, hypoechoic parotid gland, the hypoechoic muscle (dorsal part), and the echoic skin as an outer layer (Figure 3b). Calipers were used to measure the sonographically visible length of the bulla wall in this position (a straight echoic line on the lateral edge of the tympanic bulla).

### 3.2. Ultrasonography of Tympanic Bulla and Surrounding Structures in Live Llamas and Alpacas and Measurement of Sonographically Visible Length of Bulla Wall

This field study was performed on a convenience sample of 71 animals (alpacas: *n* = 50; llamas: *n* = 21) older than 1 year (mean: 8 years) and originating from six different herds. The youngest animal was 1 year old (study inclusion criteria), the oldest llama was 19 years old, and the oldest alpaca was 17 years old. In alpacas and llamas, more females (alpacas: *n* = 29; llamas: *n* = 13) than males (including intact and castrated males) (alpacas: *n* = 21; llamas: *n* = 8) were examined. Body condition scores varied between 2.0 and 3.5 in alpacas and between 2.5 and 3.5 in llamas (Table 1 and Table 2).

**Figure 4:**
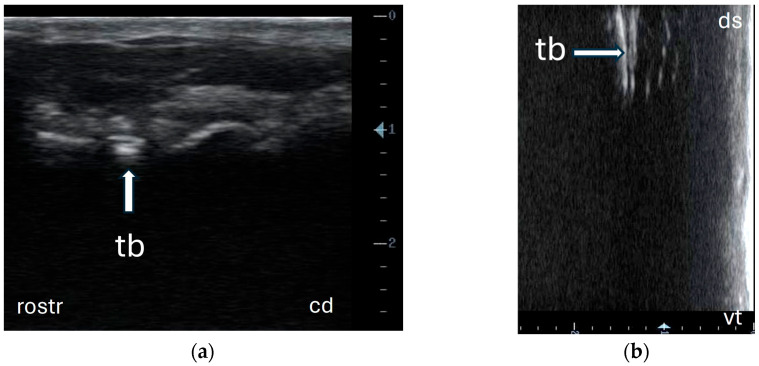
Sonogram of tympanic bulla in a live alpaca without clipping the fleece in the region of interest. (**a**) sonogram of tympanic bulla (arrow) in rostrocaudal probe position using lateral approach ventral to base of ear: tympanic bulla (tb), caudal (cd), and rostral (rostr); (**b**) sonogram of tympanic bulla (arrow) in dorsoventral probe position using lateral approach ventral to base of ear: tympanic bulla (tb), dorsal (ds), and ventral (vt).

In the field, the palpation of anatomical landmarks was performed before the ultrasonographic examination for optimal probe positioning, such as the vertical mandibular ramus with its condylar and subcondylar process and the tympanic bulla, which is located directly ventral to the base of the ear. The sonographic examination protocol, established according to the results of ultrasonography of the bulla in the cadaver specimen, started with a rostrocaudal probe position, followed by a dorsoventral probe position, while also measuring the tympanic bulla’s visible length (Figure 4a,b).

All animals tolerated the ultrasonographic examination well. The examination was discontinued in none of the animals. It was possible to sonographically identify the tympanic bulla and surrounding structures using a lateral approach with two probe positions in all the llamas and alpacas examined. The sonogram did not differ from the ultrasonographic appearance in the cadaver specimens. Moreover, measuring the length of the lateral bulla wall (a straight echoic line) in the dorsoventral probe position was feasible in all animals (Table 3). The visible length (cm) of the lateral bulla wall was significantly longer in llamas than in alpacas (*p* = 0.002). In both animal species, no correlation was observed between the BCS and length of the lateral tympanic bulla wall. The correlation between length, gender, and age showed that older female alpacas had a significantly shorter length (r = −0.380; *p* = 0.042). In llamas, no significant correlation was observed between the length of the lateral tympanic bulla wall, gender, and age.

**Figure 5:**
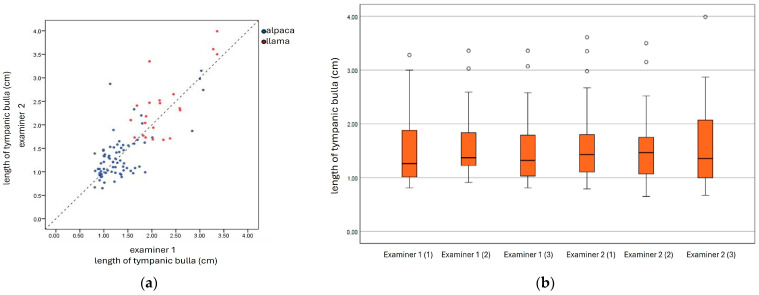
Comparison of measurement results (sonographically visible length of lateral tympanic bulla wall) from two examiners (Examiners 1 and 2): (**a**) the correlation between the two examiners showed a significant result, the dashed line represents the regression line; (**b**) box plots representing the measuring results (Examiners 1 and 2) of three repeated examinations (1, 2, and 3) in each animal (*n* = 16), the dots represent the outliers.

The calculation of the intra-observer reliability between the two examiners yielded a significantly good agreement (examiner 1: ICC = 0.86; examiner 2: ICC = 0.85). The correlation was significant between the two examiners, both in llamas and alpacas (llamas: r = 0.73; alpacas: r = 0.69; *p* < 0.001) (Figure 5).

## 4. Discussion

Ultrasonography, a non-invasive diagnostic imaging technique, is used to detect soft-tissue changes and examine bony surfaces [11,12]. As abscessation, osteolysis, and sequestration of the bulla wall often develop in cases of chronic otitis media in SACs [1,4,7,9], ultrasonography was used as a diagnostic tool. Successful use of this technique in diseased animals requires knowledge of the physiological sonographic appearance of structures and scanning methods. Therefore, this study aimed to establish a scanning technique for visualizing the tympanic bulla and surrounding structures and describe its normal sonographic appearance in SACs.

Sonographic examination of the tympanic bulla has already been performed in other animal species. In general, the use of high-frequency linear probes between 7.5 and 12 MHz is recommended, since the tympanic bulla is a superficial structure [13,15,16,18,19,20]. In dogs, ultrasonographic examinations of the tympanic bulla also revealed good results with lower frequency probes (3.5 and 6.5 MHz), showing the best results using a 6.5 MHz curvilinear transducer [13]. Our results showed that using a 59 × 10 mm linear probe with a 10 MHz frequency allowed adequate positioning in the acoustic window and produced a good image resolution of the tympanic bulla and soft tissue (the parotid gland and muscle layer) via a lateral approach in alpacas and llamas. The size and form of the probe play an important role in obtaining an ideal positioning since the acoustic window has small dimensions in the area of interest. The authors assumed that rectal probes may not be applicable in camelids due to their larger dimension that reduces maneuverability. In a study on calves, a rectal probe was evaluated as a probe with poorer handling ability and image quality when visualizing the tympanic bulla and surrounding structures [16].

Different probes and probe positions have been described in different animal species. While ultrasonographic examination of the tympanic bulla in dogs works with a lateral and a ventral approach, either on a standing or recumbent animal [13], cats and rabbits have been examined only using a ventral approach due to anatomical reasons [15,20,21]. In calves and donkeys, only the lateral approach provided an adequate acoustic window for displaying the bulla [16,19,22]. The lateral approach was chosen and evaluated in this study due to the applicable probe positioning in the region of interest, showing good results in the rostrocaudal and dorsoventral probe positions. Extending the head more to the contralateral side in live animals did not lead to the enlargement of the acoustic window. In a study involving dogs, it was necessary to extend the position of the head and neck significantly more for live animals than cadaver specimens to visualize the tympanic bulla in both lateral and ventral probe positions [13].

Ultrasonography, using a lateral approach and applying moderate pressure with the transducer, was well tolerated by all camelids without sedation in this study. Simply holding the animal’s head by one person was sufficient. Unlike with other species, clipping the fleece was unnecessary. Using alcohol as a contact medium provided optimal images, which was already demonstrated on this animal species in a study involving the ultrasonographic examination of abdominal organs [28]. Avoiding shearing in SACs seems to be beneficial, since camelids are very sensitive to stress caused by the loud noise of shearing machines.

From the authors’ point of view, the ultrasound examination protocol of the bulla in camelids should start with the rostrocaudal probe position, since this ultrasound beam allows for better identification of the bulla and the surrounding structures, followed by the dorsoventral probe position, which allows for the evaluation of the bony surface. Manual palpation for the identification of anatomical landmarks for optimal probe positioning before the ultrasonographic examination is necessary.

In our study, the lateral tympanic bulla wall was displayed as a smooth echoic line with acoustic shadowing and reverberation artifacts, which is consistent with the physical behavior of ultrasound waves when encountering a gas-filled structure [11,12]. This ultrasonographic image of the bulla wall was comparable to other animal species, where the bulla also appeared as a hyperechoic interface creating “dirty” acoustic shadowing and masking deeper structures [13,15,16,17,19,20].

A fluid transmits most of the echoes and, therefore, helps attain a better visualization of the examined structures [11]. In studies with small animals, experimentally filling the bulla with a fluid made the trabeculations of the far wall visible, aiding identification [13,23,24]. The penetration of the tympanic membrane was necessary to fill the bulla with fluid. The authors stated that this situation is advantageous, since in animals with otitis media, the accumulation of fluid appears as a sign of inflammation, and pathological changes, such as interrupted or irregular trabeculae or the deformation of the bulla wall, are visible [16,19,22,23]. Fluid within the bulla was even detected in healthy calf cadavers (mature fetuses) and live healthy calves aged between 2 and 10 weeks, which was attributed by the authors to low ossification levels [16]. No bulla fluid was found in the animals of our study, where only llamas and alpacas older than 1 year were examined. For the detection of bulla fluid, ultrasonography revealed a better sensitivity and specificity in small animal cadavers compared with radiography [15,17,21,23].

In our study, it was not possible to penetrate the tympanic membrane and experimentally fill the bulla with fluid in the camelid cadavers due to the specific shape of the external ear canal (the perpendicular ventral part) as well as the inner honeycomb-structured tympanic bulla.

For the sonographic visualization of the content of the tympanic bulla, the thickness of the osseous bulla wall affects the degree to which echoes penetrate the bone. In rabbits, the ultrasonographic examination of the bulla is, therefore, performed using a ventral approach due to the thicker lateral wall compared with the ventral bulla wall [15]. To the authors’ knowledge, no information about the thickness of the osseous tympanic bulla wall in healthy camelids is available.

In camelids with chronic otitis media, abscessation can develop and lead to the lysis of the inner honeycomb bone lamellae [1,2,5,8]. The authors believe that this condition can cause, in a late stage of disease, the thinning of the tympanic bulla wall and facilitate beam penetration, aiding diagnosis. The presence of bone lysis and sequestration has been described in chronically diseased camelids [1,2,5,8]. Since these pathological changes might be detected via ultrasonography, displayed as a discontinuity or irregularity of the bulla wall, we considered looking at the sonographic appearance of the tympanic bulla wall surface as well as the length of the visible part. Therefore, the sonographically visible part of the tympanic bulla wall was measured, comparing llamas and alpacas, as well as males and females, to have an idea about normal reference values. Llamas had longer tympanic bullae than alpacas, likely due to their larger body size. In alpacas, older females had shorter tympanic bullae, though the reason for this was unclear. The BCS did not affect the length of the bulla wall and, therefore, did not appear to influence the size of the acoustic window or the available space for transducer positioning. The number of animals observed should be considered when interpreting these findings.

Looking at the results of the correlation between the two examiners and the intra-observer reliability in this study, it can be concluded that ultrasonographic visualization of the tympanic bulla and measurement in healthy camelids is a reliable method. Nevertheless, our results dealing with the length of the tympanic bulla wall showed a wide range, which questions this parameter as a reliable indicator for detecting diseased animals.

This study had limitations that could be addressed in future research. First, animals younger than one year were excluded to gather insights into the feasibility of ultrasonographic imaging to visualize the tympanic bulla in older camelids before examining younger animals. Second, only a small number of llamas were examined.

## 5. Conclusions

The results of this study indicate that ultrasonography is a feasible technique for visualizing the tympanic bulla and surrounding structures in healthy camelids with a lateral approach using high-frequency linear probes.

Since otitis media in camelids can significantly impair the animal’s general condition and may even lead to death if treatment is delayed or absent, an early and accurate diagnosis of the disease is important. In such cases, a non-invasive diagnostic imaging technique that can be performed in the field using portable devices without sedation of the animal would be highly advantageous for diagnosis and prognosis. Further studies are required to evaluate the diagnostic potential of ultrasonography in diseased camelids before implementation in clinical practice.

## Figures and Tables

**Table 1:** Age, gender, and body condition scores (BCS) of examined llamas (N), given as mean (M), standard deviation (SD), minimum (MIN), and maximum (MAX).

Llamas: Measured Parameter	Gender	N	M	SD	Min.	Max.
Age	Male	8	10.4	6.36	2	19
	Female	12 *	8.8	8.75	3	15
BCS	Male	8	3.1	0.17	3	3.5
	Female	13	2.8	0.24	2.5	3

* = no age was available for one female llama.

**Table 2:** Age, gender, and body condition scores (BCS) of examined alpacas (N), given as mean (M), standard deviation (SD), minimum (min.), and maximum (max.).

Alpacas: Measured Parameter	Gender	N	M	SD	Min.	Max.
Age	Male	21	7.4	3.62	1	17
	Female	29	7.5	4.21	1	13
BCS	Male	21	2.9	0.35	2	3
	Female	29	2.7	0.44	2	3.5

**Table 3:** Sonographically measured length of lateral tympanic bulla wall (L-TB) using lateral approach in male and female llamas and alpacas (N), given as mean (M), standard deviation (SD), minimum (min.), and maximum (max.).

L-TB (cm)	Gender	N	M (cm)	SD (cm)	Min. (cm)	Max. (cm)
Llamas	Male	8	1.53	0.36	1.15	2.24
	Female	13	1.80	0.51	1.16	3.28
Alpacas	Male	21	1.43	0.46	0.63	2.44
	Female	29	1.36	0.30	0.64	1.88

cm = length in centimeters of lateral tympanic bulla wall.

## Data Availability

The data that support the findings of this study are available from the corresponding author upon reasonable request.

## Abbreviations

The following abbreviations were used in this manuscript:

M	Mean
BCS	Body condition score
CT	Computed tomography
GSP	Good scientific practice
ICC	Intraclass correlation coefficient
MAX	Maximum
MHz	Mega Hertz
MIN	Minimum
SACs	South American camelids
SD	Standard deviation
